# Supplementary material for: The Prognostic Significance of Epidermal Growth Factor Receptor Amplification and Epidermal Growth Factor Receptor Variant III Mutation in Glioblastoma: A Systematic Review and Meta-Analysis with Implications for Targeted Therapy
Source: Int J Mol Sci. 2025 Apr 9;26(8):3539. doi: 10.3390/ijms26083539 (PMC12027172; doi:10.3390/ijms26083539)
Supplement: Supplementary file 1 [file ijms-26-03539-s001.zip › ijms-3538166-supplementary.pdf]

Prognostic Significance of EGFR Amplification and EGFRvIII Mutation in Glioblastoma: A Systematic Review and Meta-Analysis with Implications for Targeted Therapy

Supplementary Digital Files

Content

Table S1. PRISMA Checklist
Table S2. Detailed Search Strategy and Keywords for Literature Inclusion
Figure S1-S8.

Table S1. PRISMA Checklist

Section and Topic	Item #	Checklist item	Location where item is reported
TITLE			
Title	1	Identify the report as a systematic review.	1
ABSTRACT			
Abstract	2	See the PRISMA 2020 for Abstracts checklist.	1
INTRODUCTION			
Rationale	3	Describe the rationale for the review in the context of existing knowledge.	2
Objectives	4	Provide an explicit statement of the objective(s) or question(s) the review addresses.	2, 3
METHODS			
Eligibility criteria	5	Specify the inclusion and exclusion criteria for the review and how studies were grouped for the syntheses.	3
Information sources	6	Specify all databases, registers, websites, organisations, reference lists and other sources searched or consulted to identify studies. Specify the date when each source was last searched or consulted.	3
Search strategy	7	Present the full search strategies for all databases, registers and websites, including any filters and limits used.	3
Selection process	8	Specify the methods used to decide whether a study met the inclusion criteria of the review, including how many reviewers screened each record and each report retrieved, whether they worked independently, and if applicable, details of automation tools used in the process.	3
Data collection process	9	Specify the methods used to collect data from reports, including how many reviewers collected data from each report, whether they worked independently, any processes for obtaining or confirming data from study investigators, and if applicable, details of automation tools used in the process.	3
Data items	10a	List and define all outcomes for which data were sought. Specify whether all results that were compatible with each outcome domain in each study were sought (e.g. for all measures, time points, analyses), and if not, the methods used to decide which results to collect.	3
	10b	List and define all other variables for which data were sought (e.g. participant and intervention characteristics, funding sources). Describe any assumptions made about any missing or unclear information.	3
Study risk of bias assessment	11	Specify the methods used to assess risk of bias in the included studies, including details of the tool(s) used, how many reviewers assessed each study and whether they worked independently, and if applicable, details of automation tools used in the process.	3, 4
Effect measures	12	Specify for each outcome the effect measure(s) (e.g. risk ratio, mean difference) used in the synthesis or presentation of results.	4
Synthesis methods	13a	Describe the processes used to decide which studies were eligible for each synthesis (e.g. tabulating the study intervention characteristics and comparing against the planned groups for each synthesis (item #5)).	4, 5
	13b	Describe any methods required to prepare the data for presentation or synthesis, such as handling of missing summary statistics, or data conversions.	3
	13c	Describe any methods used to tabulate or visually display results of individual studies and syntheses.	3, 4
	13d	Describe any methods used to synthesize results and provide a rationale for the choice(s). If meta-analysis was performed, describe the model(s), method(s) to identify the presence and extent of statistical heterogeneity, and software package(s) used.	4
	13e	Describe any methods used to explore possible causes of heterogeneity among study results (e.g. subgroup analysis, meta-regression).	4
	13f	Describe any sensitivity analyses conducted to assess robustness of the synthesized results.	4
Reporting bias assessment	14	Describe any methods used to assess risk of bias due to missing results in a synthesis (arising from reporting biases).	4
Certainty assessment	15	Describe any methods used to assess certainty (or confidence) in the body of evidence for an outcome.	3, 4

Table S1. PRISMA Checklist

Section and Topic	Item #	Checklist item	Location where item is reported
RESULTS			
Study selection	16a	Describe the results of the search and selection process, from the number of records identified in the search to the number of studies included in the review, ideally using a flow diagram.	4, 5
	16b	Cite studies that might appear to meet the inclusion criteria, but which were excluded, and explain why they were excluded.	4, 5
Study characteristics	17	Cite each included study and present its characteristics.	4-9
Risk of bias in studies	18	Present assessments of risk of bias for each included study.	9, 10
Results of individual studies	19	For all outcomes, present, for each study: (a) summary statistics for each group (where appropriate) and (b) an effect estimate and its precision (e.g. confidence/credible interval), ideally using structured tables or plots.	11-17
Results of syntheses	20a	For each synthesis, briefly summarise the characteristics and risk of bias among contributing studies.	11-17
	20b	Present results of all statistical syntheses conducted. If meta-analysis was done, present for each the summary estimate and its precision (e.g. confidence/credible interval) and measures of statistical heterogeneity. If comparing groups, describe the direction of the effect.	11-17
	20c	Present results of all investigations of possible causes of heterogeneity among study results.	11-17
	20d	Present results of all sensitivity analyses conducted to assess the robustness of the synthesized results.	16
Reporting biases	21	Present assessments of risk of bias due to missing results (arising from reporting biases) for each synthesis assessed.	16
Certainty of evidence	22	Present assessments of certainty (or confidence) in the body of evidence for each outcome assessed.	16
DISCUSSION			
Discussion	23a	Provide a general interpretation of the results in the context of other evidence.	17-20
	23b	Discuss any limitations of the evidence included in the review.	17-20
	23c	Discuss any limitations of the review processes used.	17-20
	23d	Discuss implications of the results for practice, policy, and future research.	17-20
OTHER INFORMATION			
Registration and protocol	24a	Provide registration information for the review, including register name and registration number, or state that the review was not registered.	3
	24b	Indicate where the review protocol can be accessed, or state that a protocol was not prepared.	3
	24c	Describe and explain any amendments to information provided at registration or in the protocol.	-
Support	25	Describe sources of financial or non-financial support for the review, and the role of the funders or sponsors in the review.	21
Competing interests	26	Declare any competing interests of review authors.	21
Availability of data, code and other materials	27	Report which of the following are publicly available and where they can be found: template data collection forms; data extracted from included studies; data used for all analyses; analytic code; any other materials used in the review.	21

Table S2. Detailed search strategy and parameters used for study selection.

Database	Query (as of November 2024)	Items found
**PubMed**	(("Glioblastoma"[MeSH] OR glioblastoma*[Title/Abstract] OR GBM[Title/Abstract]) AND (("EGFRvIII"[Title/Abstract] OR "EGFR variant III"[Title/Abstract] OR "epidermal growth factor receptor variant III"[Title/Abstract]) OR ("EGFR mutation"[Title/Abstract] OR "epidermal growth factor receptor mutation"[Title/Abstract] OR "EGFR mutations"[Title/Abstract])) AND (("Cohort Studies"[MeSH] OR cohort[Title/Abstract] OR prospective[Title/Abstract] OR retrospective[Title/Abstract]) OR ("Case-Control Studies"[MeSH] OR case-control[Title/Abstract] OR matched-pairs[Title/Abstract])))	85
**Web of Science (All databases)**	TS=((("EGFRvIII" OR "epidermal growth factor receptor variant III" OR "EGFR variant III") OR ("EGFR amplification" OR "EGFR-amplified" OR "EGFR positive" OR "EGFR+")) AND ("glioblastoma" OR "glioblastoma multiforme" OR "GBM" OR "high-grade glioma") AND ("survival" OR "overall survival" OR "progression-free survival" OR "PFS" OR "OS") AND ("case-control study" OR "cohort study" OR "retrospective study" OR "prospective study") NOT ("animal model" OR "cell line" OR "in vitro"))	46
**Scopus**	TITLE-ABS-KEY ("EGFRvIII" OR "epidermal growth factor receptor variant III" OR "EGFR variant III") AND TITLE-ABS-KEY ("glioblastoma" OR "glioblastoma multiforme" OR "GBM" OR "high-grade glioma") AND TITLE-ABS-KEY ("survival" OR "overall survival" OR "progression-free survival" OR "PFS" OR "OS") AND TITLE-ABS-KEY ("case-control study" OR "cohort study" OR "retrospective study" OR "prospective study") AND TITLE-ABS-KEY ("hazard ratio" OR "risk ratio" OR "odds ratio" OR "HR" OR "RR" OR "OR") AND NOT TITLE-ABS-KEY ("animal model" OR "cell line" OR "in vitro")	20

a

Study or Subgroup	log[Hazard Ratio]	SE	Weight	Hazard Ratio IV, Fixed, 95% CI
Aldape KD 2004	0.0862	0.2407	4.9%	1.09 [0.68, 1.75]
Chiesa S 2022	1.307	0.8858	0.4%	3.70 [0.65, 20.97]
Dundar 2024	−0.2107	0.4429	1.5%	0.81 [0.34, 1.93]
Faulkner 2015	0.1989	0.4065	1.7%	1.22 [0.55, 2.71]
Friedman 2023	0.5933	0.2404	5.0%	1.81 [1.13, 2.90]
Heimberger 2005	0.2927	0.2204	5.9%	1.34 [0.87, 2.06]
Jose 2020	−0.1863	0.4262	1.6%	0.83 [0.36, 1.91]
Lassman 2023	−0.0513	0.1558	11.8%	0.95 [0.70, 1.29]
Lauretti L 2022	−0.207	0.1142	22.0%	0.81 [0.65, 1.02]
Lv S 2012	0.5247	0.388	1.9%	1.69 [0.79, 3.62]
Montano 2011	−0.8164	0.3821	2.0%	0.44 [0.21, 0.93]
Navarro 2020	0.3577	0.1774	9.1%	1.43 [1.01, 2.02]
Nozawa 2019	0.0296	0.3896	1.9%	1.03 [0.48, 2.21]
Pelloski 2007	1.2149	0.3373	2.5%	3.37 [1.74, 6.53]
Salvati 2020	0.5247	0.2886	3.4%	1.69 [0.96, 2.98]
Sepúlveda-Sánchez 2017	0.4824	0.3231	2.7%	1.62 [0.86, 3.05]
Struve 2020	−0.2485	0.1512	12.5%	0.78 [0.58, 1.05]
Viana 2008	−0.312	0.8779	0.4%	0.73 [0.13, 4.09]
Weller 2014	−0.2877	0.2721	3.9%	0.75 [0.44, 1.28]
Yang K 2019	−0.0899	0.2409	4.9%	0.91 [0.57, 1.47]
**Total (95% CI)**			**100.0%**	**1.04 [0.94, 1.16]**

Heterogeneity: $Chi^2 = 46.63$, df = 19 (P = 0.0004); $I^2 = 59\%$
Test for overall effect: Z = 0.80 (P = 0.42)

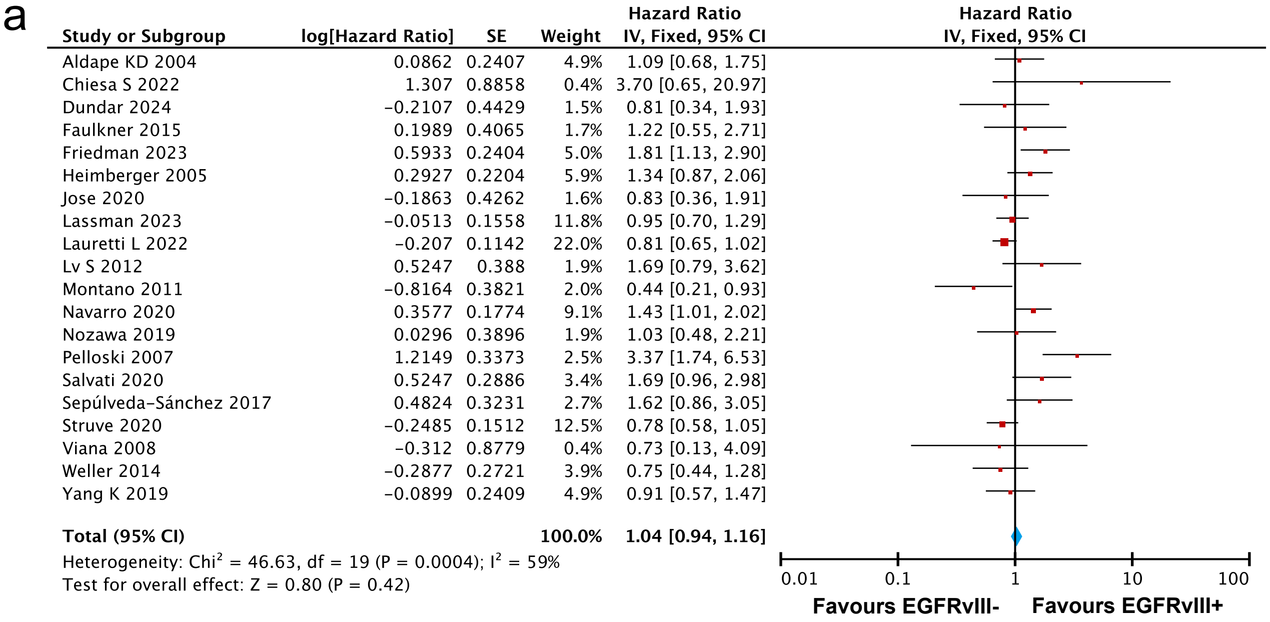

b

Study or Subgroup	log[Hazard Ratio]	SE	Weight	Hazard Ratio IV, Fixed, 95% CI
Abdullah 2015	0.793	0.3749	3.1%	2.21 [1.06, 4.61]
Alimohamadi M 2024	−0.4463	0.3079	4.6%	0.64 [0.35, 1.17]
Armocida 2019	−0.1054	0.166	15.8%	0.90 [0.65, 1.25]
Binder 2018	0.2311	0.1387	22.6%	1.26 [0.96, 1.65]
Dundar 2024	0.4318	0.2973	4.9%	1.54 [0.86, 2.76]
Faulkner 2015	0.0296	0.379	3.0%	1.03 [0.49, 2.17]
Friedman 2023	1.3661	0.6819	0.9%	3.92 [1.03, 14.92]
Hoffman 2019	0.8329	0.5517	1.4%	2.30 [0.78, 6.78]
Lasica 2021	0.7839	0.3607	3.3%	2.19 [1.08, 4.44]
Pulcini S 2024	−0.0305	0.2451	7.2%	0.97 [0.60, 1.57]
Synhaeve 2018	−0.1165	0.1682	15.4%	0.89 [0.64, 1.24]
Vaios 2017	0.5761	0.3053	4.7%	1.78 [0.98, 3.24]
Viana 2008	0.0705	0.9491	0.5%	1.07 [0.17, 6.89]
Yang 2022	0.5128	0.1859	12.6%	1.67 [1.16, 2.40]
**Total (95% CI)**			**100.0%**	**1.20 [1.06, 1.37]**

Heterogeneity: $Chi^2 = 26.75$, df = 13 (P = 0.01); $I^2 = 51\%$
Test for overall effect: Z = 2.79 (P = 0.005)

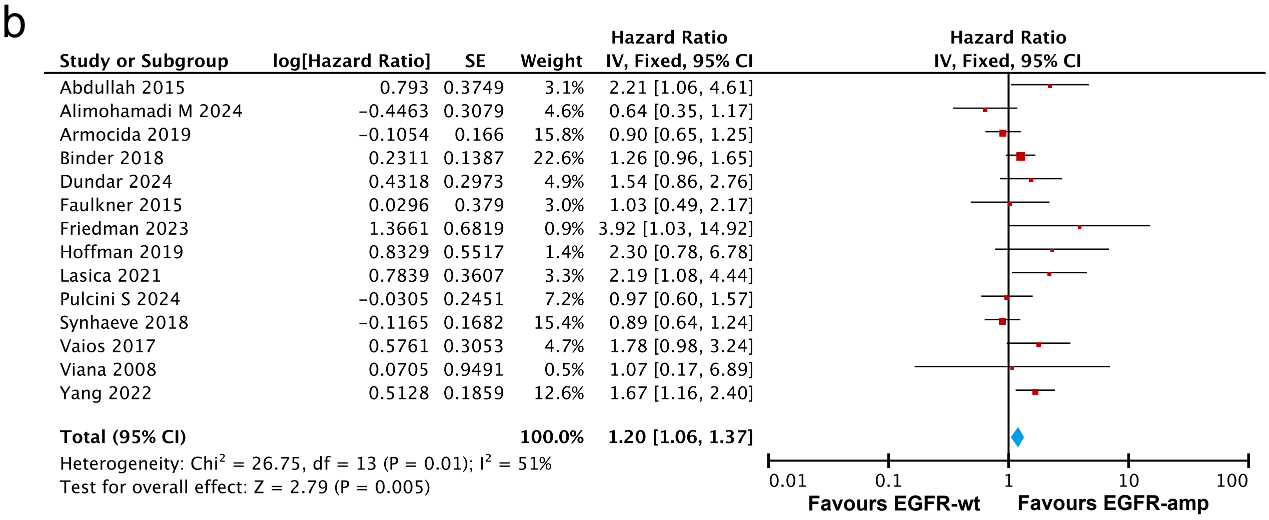

Figure S1. Fixed-effects model analysis of the association between EGFRvIII mutation (a) and EGFR amplification (b) with OS in GBM patients.

a

Study or Subgroup	log[Hazard Ratio]	SE	Weight	Hazard Ratio IV, Fixed, 95% CI
Chiesa S 2022	0.8134	0.6224	3.0%	2.26 [0.67, 7.64]
Jose 2020	−0.0834	0.4787	5.0%	0.92 [0.36, 2.35]
Lassman 2023	−0.3011	0.1332	64.5%	0.74 [0.57, 0.96]
Lv S 2012	−0.462	0.4716	5.1%	0.63 [0.25, 1.59]
Sepúlveda-Sánchez 2017	0.1222	0.3674	8.5%	1.13 [0.55, 2.32]
Weller 2014	0.6098	0.2862	14.0%	1.84 [1.05, 3.22]
**Total (95% CI)**			**100.0%**	**0.90 [0.73, 1.11]**

Heterogeneity: $Chi^2 = 11.54$, df = 5 (P = 0.04); $I^2 = 57\%$
Test for overall effect: Z = 0.96 (P = 0.34)

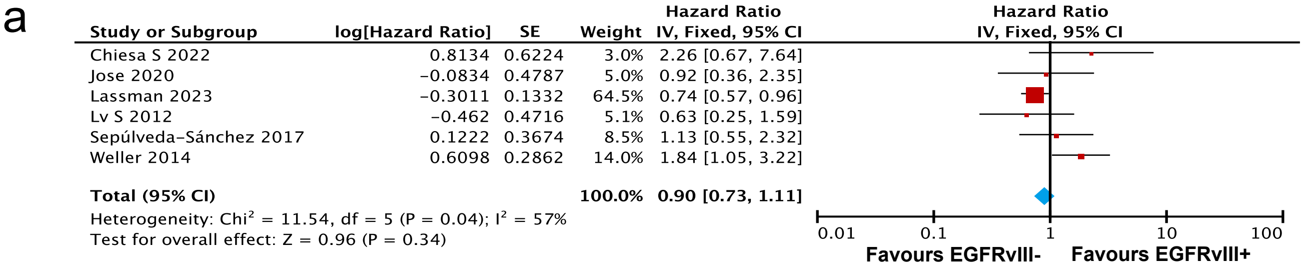

b

Study or Subgroup	log[Hazard Ratio]	SE	Weight	Hazard Ratio IV, Fixed, 95% CI
Alimohamadi M 2024	−0.4463	0.2936	16.5%	0.64 [0.36, 1.14]
Hovinga 2019	1.3863	0.458	6.8%	4.00 [1.63, 9.82]
Lasica 2021	0.1823	0.1943	37.8%	1.20 [0.82, 1.76]
Limam 2019	−0.2332	1.1096	1.2%	0.79 [0.09, 6.97]
Yang 2022	0.1823	0.1943	37.8%	1.20 [0.82, 1.76]
**Total (95% CI)**			**100.0%**	**1.17 [0.92, 1.48]**

Heterogeneity: $Chi^2 = 11.58$, df = 4 (P = 0.02); $I^2 = 65\%$
Test for overall effect: Z = 1.30 (P = 0.19)

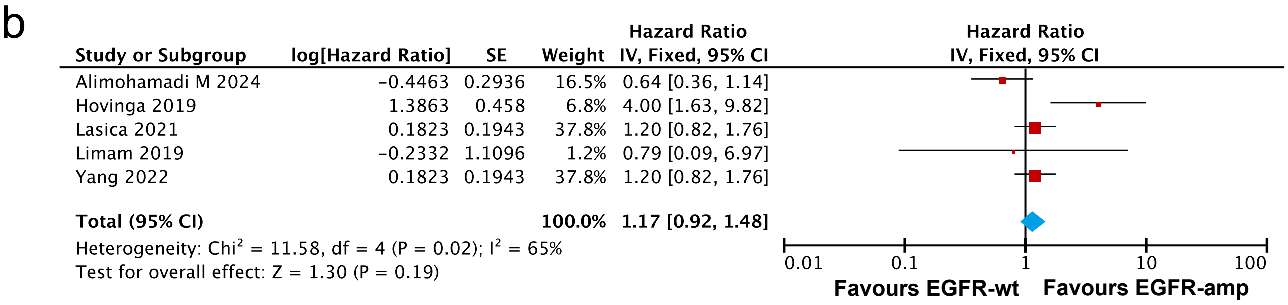

Figure S2. Fixed-effects model analysis of the association between EGFRvIII mutation (a) and EGFR amplification (b) with progression-free survival (PFS) in GBM patients.

a

Study or Subgroup	log[Hazard Ratio]	SE	Weight	Hazard Ratio IV, Fixed, 95% CI	Hazard Ratio IV, Fixed, 95% CI
**1.5.1 Asian**					
Jose 2020	−0.1863	0.4262	2.1%	0.83 [0.36, 1.91]	
Nozawa 2019	0.0296	0.3896	2.5%	1.03 [0.48, 2.21]	
Yang K 2019	−0.0899	0.2409	6.5%	0.91 [0.57, 1.47]	
**Subtotal (95% CI)**			**11.1%**	**0.92 [0.64, 1.32]**	
Heterogeneity: $Chi^2 = 0.14$, df = 2 (P = 0.93); $I^2 = 0\%$					
Test for overall effect: Z = 0.44 (P = 0.66)					
**1.5.2 American**					
Aldape KD 2004	0.0862	0.2407	6.5%	1.09 [0.68, 1.75]	
Dundar 2024	−0.2107	0.4429	1.9%	0.81 [0.34, 1.93]	
Friedman 2023	0.5933	0.2404	6.6%	1.81 [1.13, 2.90]	
Heimberger 2005	0.2927	0.2204	7.8%	1.34 [0.87, 2.06]	
Pelloski 2007	1.2149	0.3373	3.3%	3.37 [1.74, 6.53]	
**Subtotal (95% CI)**			**26.1%**	**1.49 [1.17, 1.88]**	
Heterogeneity: $Chi^2 = 10.32$, df = 4 (P = 0.04); $I^2 = 61\%$					
Test for overall effect: Z = 3.30 (P = 0.0010)					
**1.5.3 European**					
Chiesa S 2022	1.307	0.8858	0.5%	3.70 [0.65, 20.97]	
Faulkner 2015	0.1989	0.4065	2.3%	1.22 [0.55, 2.71]	
Lauretti L 2022	−0.207	0.1142	29.0%	0.81 [0.65, 1.02]	
Lv S 2012	0.5247	0.388	2.5%	1.69 [0.79, 3.62]	
Montano 2011	−0.8164	0.3821	2.6%	0.44 [0.21, 0.93]	
Navarro 2020	0.3577	0.1774	12.0%	1.43 [1.01, 2.02]	
Salvati 2020	0.5247	0.2886	4.5%	1.69 [0.96, 2.98]	
Sepúlveda-Sánchez 2017	0.4824	0.3231	3.6%	1.62 [0.86, 3.05]	
Viana 2008	−0.312	0.8779	0.5%	0.73 [0.13, 4.09]	
Weller 2014	−0.2877	0.2721	5.1%	0.75 [0.44, 1.28]	
**Subtotal (95% CI)**			**62.7%**	**1.02 [0.87, 1.18]**	
Heterogeneity: $Chi^2 = 22.89$, df = 9 (P = 0.006); $I^2 = 61\%$					
Test for overall effect: Z = 0.22 (P = 0.82)					
**Total (95% CI)**			**100.0%**	**1.11 [0.99, 1.25]**	
Heterogeneity: $Chi^2 = 41.51$, df = 17 (P = 0.0008); $I^2 = 59\%$					
Test for overall effect: Z = 1.72 (P = 0.09)					
Test for subgroup differences: $Chi^2 = 8.16$, df = 2 (P = 0.02), $I^2 = 75.5\%$					

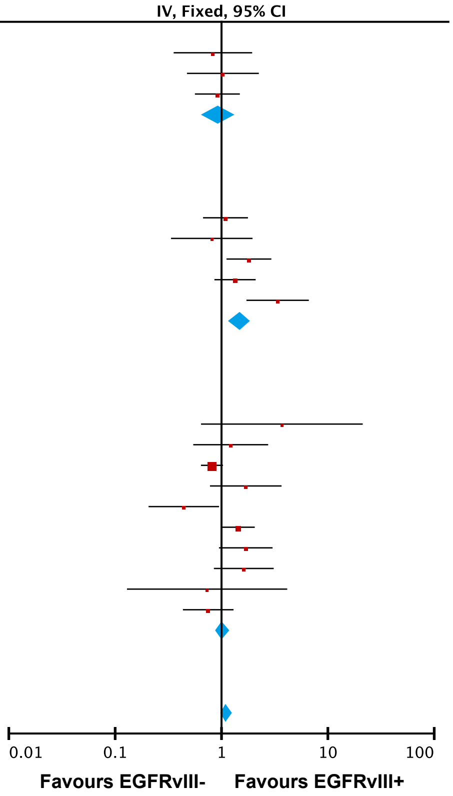

b

Study or Subgroup	log[Hazard Ratio]	SE	Weight	Hazard Ratio IV, Random, 95% CI	Hazard Ratio IV, Random, 95% CI
**2.5.1 American**					
Abdullah 2015	0.793	0.3749	6.0%	2.21 [1.06, 4.61]	
Binder 2018	0.2311	0.1387	15.0%	1.26 [0.96, 1.65]	
Dundar 2024	0.4318	0.2973	8.1%	1.54 [0.86, 2.76]	
Friedman 2023	1.3661	0.6819	2.3%	3.92 [1.03, 14.92]	
Hoffman 2019	0.8329	0.5517	3.3%	2.30 [0.78, 6.78]	
Vaios 2017	0.5761	0.3053	7.8%	1.78 [0.98, 3.24]	
Yang 2022	0.5128	0.1859	12.6%	1.67 [1.16, 2.40]	
**Subtotal (95% CI)**			**55.3%**	**1.53 [1.28, 1.84]**	
Heterogeneity: $Tau^2 = 0.00$; $Chi^2 = 5.84$, df = 6 (P = 0.44); $I^2 = 0\%$					
Test for overall effect: Z = 4.58 (P < 0.00001)					
**2.5.2 European**					
Armocida 2019	−0.1054	0.166	13.6%	0.90 [0.65, 1.25]	
Lasica 2021	0.7839	0.3607	6.3%	2.19 [1.08, 4.44]	
Pulcini S 2024	−0.0305	0.2451	10.0%	0.97 [0.60, 1.57]	
Synhaeve 2018	−0.1165	0.1682	13.5%	0.89 [0.64, 1.24]	
Viana 2008	0.0705	0.9491	1.3%	1.07 [0.17, 6.89]	
**Subtotal (95% CI)**			**44.7%**	**1.01 [0.78, 1.30]**	
Heterogeneity: $Tau^2 = 0.02$; $Chi^2 = 5.57$, df = 4 (P = 0.23); $I^2 = 28\%$					
Test for overall effect: Z = 0.06 (P = 0.95)					
**Total (95% CI)**			**100.0%**	**1.36 [1.09, 1.68]**	
Heterogeneity: $Tau^2 = 0.06$; $Chi^2 = 22.11$, df = 11 (P = 0.02); $I^2 = 50\%$					
Test for overall effect: Z = 2.76 (P = 0.006)					
Test for subgroup differences: $Chi^2 = 6.78$, df = 1 (P = 0.009), $I^2 = 85.3\%$					

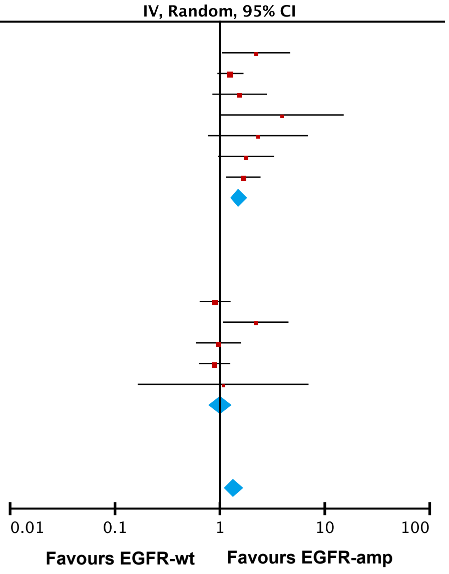

Figure S3. Subgroup analyses based on geographic populations.
(a) Fixed-effects model analysis of the association between EGFRvIII mutation and OS in GBM patients from the Americas, Asia, or Europe. (b) Random-effects model analysis of the association between EGFR amplification and OS in GBM patients from the Americas, Asia, or Europe.

Study or Subgroup	log[Hazard Ratio]	SE	Weight	Hazard Ratio IV, Random, 95% CI
**1.3.1 Newly GBM**				
Heimberger 2005	0.2927	0.2204	10.0%	1.34 [0.87, 2.06]
Lassman 2023	−0.0513	0.1558	11.6%	0.95 [0.70, 1.29]
Lauretti L 2022	−0.207	0.1142	12.5%	0.81 [0.65, 1.02]
Montano 2011	−0.8164	0.3821	6.5%	0.44 [0.21, 0.93]
Pelloski 2007	1.2149	0.3373	7.3%	3.37 [1.74, 6.53]
Sepúlveda-Sánchez 2017	0.4824	0.3231	7.6%	1.62 [0.86, 3.05]
Viana 2008	−0.312	0.8779	2.0%	0.73 [0.13, 4.09]
Weller 2014	−0.2877	0.2721	8.8%	0.75 [0.44, 1.28]
Yang K 2019	−0.0899	0.2409	9.5%	0.91 [0.57, 1.47]
**Subtotal (95% CI)**			**75.8%**	**1.04 [0.78, 1.39]**
Heterogeneity: $Tau^2 = 0.12$; $Chi^2 = 25.97$, df = 8 (P = 0.001); $I^2 = 69\%$				
Test for overall effect: Z = 0.26 (P = 0.79)				
**1.3.2 Recurremt GBM**				
Chiesa S 2022	1.307	0.8858	1.9%	3.70 [0.65, 20.97]
Friedman 2023	0.5933	0.2404	9.5%	1.81 [1.13, 2.90]
Lv S 2012	0.5247	0.388	6.4%	1.69 [0.79, 3.62]
Nozawa 2019	0.0296	0.3896	6.3%	1.03 [0.48, 2.21]
**Subtotal (95% CI)**			**24.2%**	**1.63 [1.15, 2.31]**
Heterogeneity: $Tau^2 = 0.00$; $Chi^2 = 2.44$, df = 3 (P = 0.49); $I^2 = 0\%$				
Test for overall effect: Z = 2.77 (P = 0.006)				
**Total (95% CI)**			**100.0%**	**1.16 [0.89, 1.51]**
Heterogeneity: $Tau^2 = 0.13$; $Chi^2 = 36.16$, df = 12 (P = 0.0003); $I^2 = 67\%$				
Test for overall effect: Z = 1.11 (P = 0.27)				
Test for subgroup differences: $Chi^2 = 3.80$, df = 1 (P = 0.05), $I^2 = 73.7\%$				

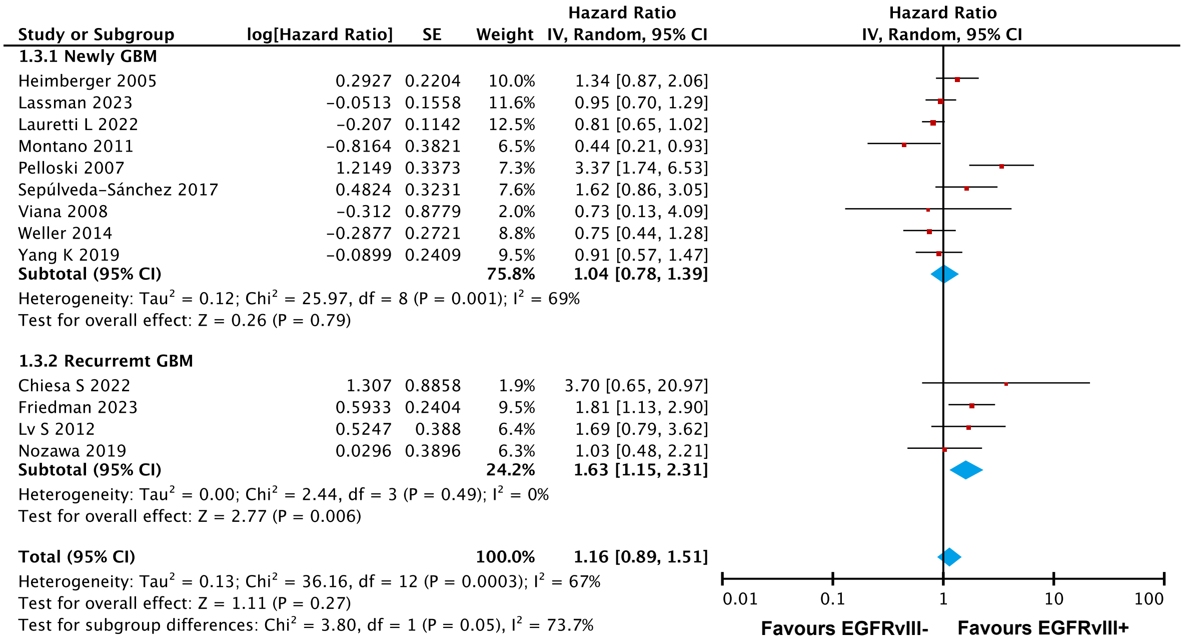

Figure S4. Subgroup analysis based on EGFRvIII mutation and OS in newly diagnosed or recurrent GBM patients. Random-effects model analysis.

a. EGFRvIII-OS

Study or Subgroup	log[Hazard Ratio]	SE	Weight	Hazard Ratio IV, Random, 95% CI
Aldape KD 2004	0.0862	0.2407	7.1%	1.09 [0.68, 1.75]
Chiesa S 2022	1.307	0.8858	1.2%	3.70 [0.65, 20.97]
Dundar 2024	−0.2107	0.4429	3.7%	0.81 [0.34, 1.93]
Faulkner 2015	0.1989	0.4065	4.1%	1.22 [0.55, 2.71]
Friedman 2023	0.5933	0.2404	7.1%	1.81 [1.13, 2.90]
Heimberger 2005	0.2927	0.2204	7.6%	1.34 [0.87, 2.06]
Jose 2020	−0.1863	0.4262	3.9%	0.83 [0.36, 1.91]
Lassman 2023	−0.0513	0.1558	9.1%	0.95 [0.70, 1.29]
Lauretti L 2022	−0.207	0.1142	10.1%	0.81 [0.65, 1.02]
Lv S 2012	0.5247	0.388	0.0%	1.69 [0.79, 3.62]
Montano 2011	−0.8164	0.3821	4.5%	0.44 [0.21, 0.93]
Navarro 2020	0.3577	0.1774	8.6%	1.43 [1.01, 2.02]
Nozawa 2019	0.0296	0.3896	4.3%	1.03 [0.48, 2.21]
Pelloski 2007	1.2149	0.3373	5.2%	3.37 [1.74, 6.53]
Salvati 2020	0.5247	0.2886	6.1%	1.69 [0.96, 2.98]
Sepúlveda-Sánchez 2017	0.4824	0.3231	0.0%	1.62 [0.86, 3.05]
Struve 2020	−0.2485	0.1512	9.2%	0.78 [0.58, 1.05]
Viana 2008	−0.312	0.8779	1.2%	0.73 [0.13, 4.09]
Weller 2014	−0.2877	0.2721	0.0%	0.75 [0.44, 1.28]
Yang K 2019	−0.0899	0.2409	7.1%	0.91 [0.57, 1.47]
**Total (95% CI)**			**100.0%**	**1.12 [0.91, 1.37]**

Heterogeneity: $Tau^2 = 0.09$; $Chi^2 = 41.73$, df = 16 (P = 0.0004); $I^2 = 62\%$
Test for overall effect: Z = 1.07 (P = 0.28)

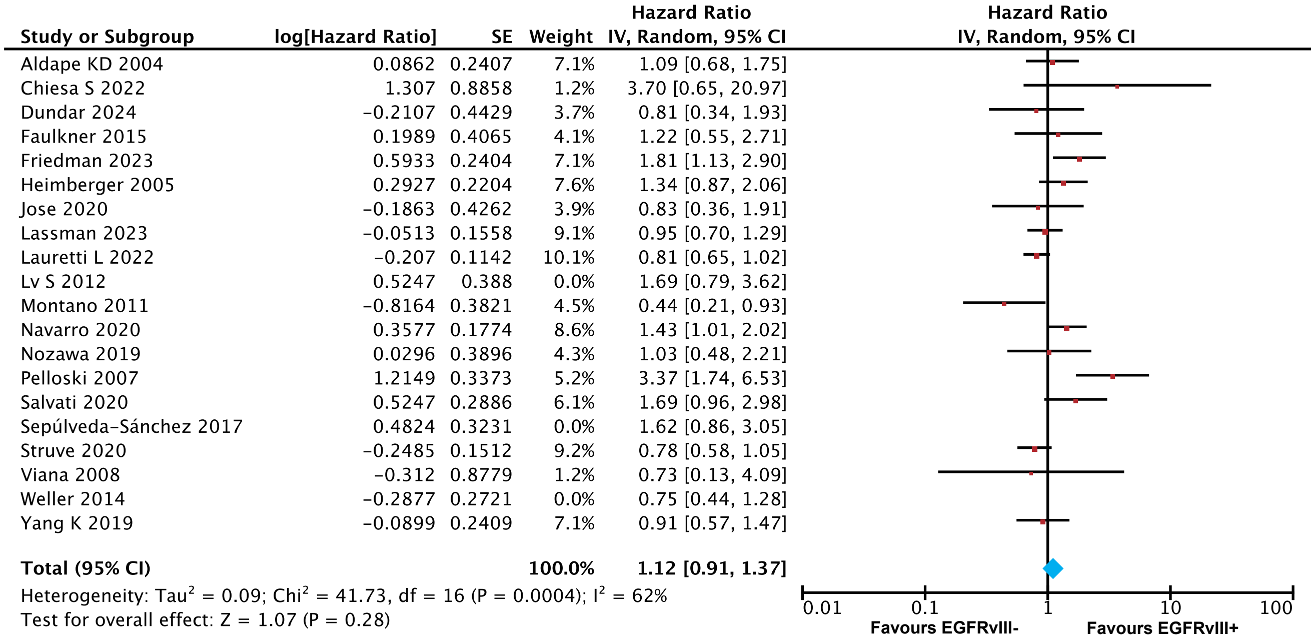

b. EGFRvIII-PFS

Study or Subgroup	log[Hazard Ratio]	SE	Weight	Hazard Ratio IV, Random, 95% CI
Chiesa S 2022	0.8134	0.6224	14.6%	2.26 [0.67, 7.64]
Jose 2020	−0.0834	0.4787	21.8%	0.92 [0.36, 2.35]
Lassman 2023	−0.3011	0.1332	63.5%	0.74 [0.57, 0.96]
Lv S 2012	−0.462	0.4716	0.0%	0.63 [0.25, 1.59]
Sepúlveda-Sánchez 2017	0.1222	0.3674	0.0%	1.13 [0.55, 2.32]
Weller 2014	0.6098	0.2862	0.0%	1.84 [1.05, 3.22]
**Total (95% CI)**			**100.0%**	**0.91 [0.54, 1.54]**

Heterogeneity: $Tau^2 = 0.09$; $Chi^2 = 3.18$, df = 2 (P = 0.20); $I^2 = 37\%$
Test for overall effect: Z = 0.34 (P = 0.73)

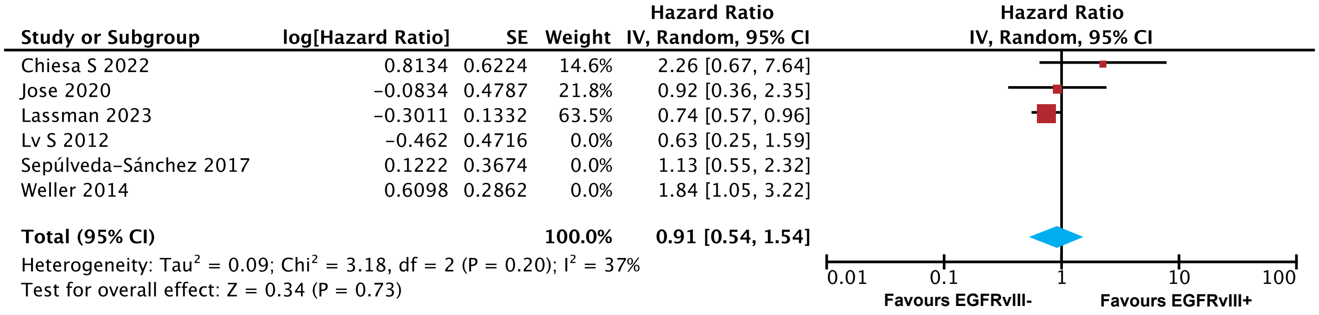

c. Subgroup analysis-EGFRvIII-Race

Study or Subgroup	log[Hazard Ratio]	SE	Weight	Hazard Ratio IV, Random, 95% CI
**1.5.1 Asian group**				
Jose 2020	−0.1863	0.4262	5.0%	0.83 [0.36, 1.91]
Nozawa 2019	0.0296	0.3896	5.6%	1.03 [0.48, 2.21]
Yang K 2019	−0.0899	0.2409	8.6%	0.91 [0.57, 1.47]
**Subtotal (95% CI)**			**19.1%**	**0.92 [0.64, 1.32]**
Heterogeneity: $Tau^2 = 0.00$; $Chi^2 = 0.14$, df = 2 (P = 0.93); $I^2 = 0\%$				
Test for overall effect: Z = 0.44 (P = 0.66)				
**1.5.2 America**				
Aldape KD 2004	0.0862	0.2407	8.6%	1.09 [0.68, 1.75]
Dundar 2024	−0.2107	0.4429	4.8%	0.81 [0.34, 1.93]
Friedman 2023	0.5933	0.2404	8.6%	1.81 [1.13, 2.90]
Heimberger 2005	0.2927	0.2204	9.1%	1.34 [0.87, 2.06]
Pelloski 2007	1.2149	0.3373	6.5%	3.37 [1.74, 6.53]
**Subtotal (95% CI)**			**37.4%**	**1.51 [1.02, 2.24]**
Heterogeneity: $Tau^2 = 0.12$; $Chi^2 = 10.32$, df = 4 (P = 0.04); $I^2 = 61\%$				
Test for overall effect: Z = 2.03 (P = 0.04)				
**1.5.3 Europe**				
Chiesa S 2022	1.307	0.8858	1.7%	3.70 [0.65, 20.97]
Faulkner 2015	0.1989	0.4065	5.3%	1.22 [0.55, 2.71]
Lauretti L 2022	−0.207	0.1142	11.5%	0.81 [0.65, 1.02]
Lv S 2012	0.5247	0.388	0.0%	1.69 [0.79, 3.62]
Montano 2011	−0.8164	0.3821	5.7%	0.44 [0.21, 0.93]
Navarro 2020	0.3577	0.1774	10.1%	1.43 [1.01, 2.02]
Salvati 2020	0.5247	0.2886	7.5%	1.69 [0.96, 2.98]
Sepúlveda-Sánchez 2017	0.4824	0.3231	0.0%	1.62 [0.86, 3.05]
Viana 2008	−0.312	0.8779	1.7%	0.73 [0.13, 4.09]
Weller 2014	−0.2877	0.2721	0.0%	0.75 [0.44, 1.28]
**Subtotal (95% CI)**			**43.4%**	**1.08 [0.73, 1.59]**
Heterogeneity: $Tau^2 = 0.14$; $Chi^2 = 17.75$, df = 6 (P = 0.007); $I^2 = 66\%$				
Test for overall effect: Z = 0.38 (P = 0.70)				
**Total (95% CI)**			**100.0%**	**1.19 [0.93, 1.51]**

Heterogeneity: $Tau^2 = 0.12$; $Chi^2 = 36.89$, df = 14 (P = 0.0008); $I^2 = 62\%$
Test for overall effect: Z = 1.39 (P = 0.16)
Test for subgroup differences: $Chi^2 = 3.31$, df = 2 (P = 0.19), $I^2 = 39.5\%$

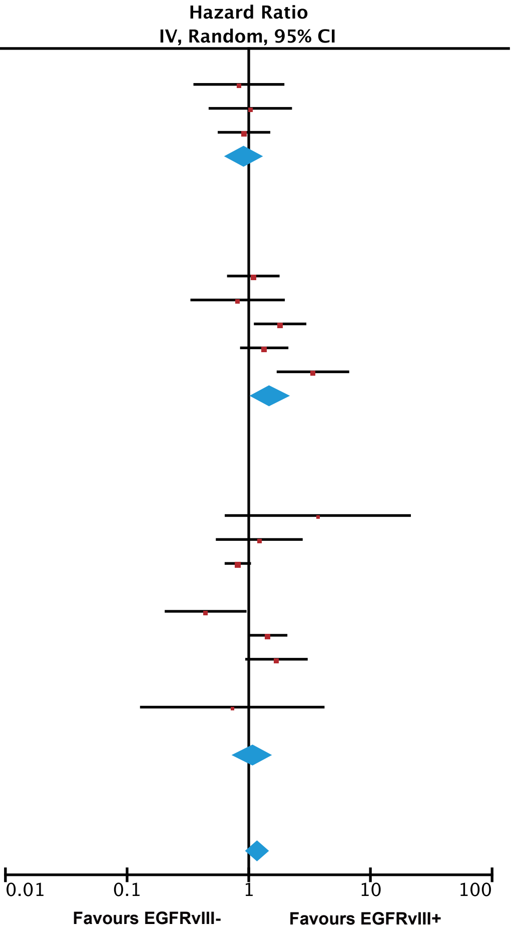

d. Subgroup analysis-EGFRvIII-Newly/Recurrent GBM

Study or Subgroup	log[Hazard Ratio]	SE	Weight	Hazard Ratio IV, Fixed, 95% CI
**1.3.1 Newly GBM**				
Heimberger 2005	0.2927	0.2204	10.4%	1.34 [0.87, 2.06]
Lassman 2023	−0.0513	0.1558	20.8%	0.95 [0.70, 1.29]
Lauretti L 2022	−0.207	0.1142	38.8%	0.81 [0.65, 1.02]
Montano 2011	−0.8164	0.3821	3.5%	0.44 [0.21, 0.93]
Pelloski 2007	1.2149	0.3373	4.4%	3.37 [1.74, 6.53]
Sepúlveda-Sánchez 2017	0.4824	0.3231	0.0%	1.62 [0.86, 3.05]
Viana 2008	−0.312	0.8779	0.7%	0.73 [0.13, 4.09]
Weller 2014	−0.2877	0.2721	0.0%	0.75 [0.44, 1.28]
Yang K 2019	−0.0899	0.2409	8.7%	0.91 [0.57, 1.47]
**Subtotal (95% CI)**			**87.3%**	**0.95 [0.82, 1.10]**
Heterogeneity: $Chi^2 = 22.51$, df = 6 (P = 0.0010); $I^2 = 73\%$				
Test for overall effect: Z = 0.67 (P = 0.50)				
**1.3.2 Recurrent GBM**				
Chiesa S 2022	1.307	0.8858	0.6%	3.70 [0.65, 20.97]
Friedman 2023	0.5933	0.2404	8.7%	1.81 [1.13, 2.90]
Lv S 2012	0.5247	0.388	0.0%	1.69 [0.79, 3.62]
Nozawa 2019	0.0296	0.3896	3.3%	1.03 [0.48, 2.21]
**Subtotal (95% CI)**			**12.7%**	**1.62 [1.10, 2.39]**
Heterogeneity: $Chi^2 = 2.43$, df = 2 (P = 0.30); $I^2 = 18\%$				
Test for overall effect: Z = 2.42 (P = 0.02)				
**Total (95% CI)**			**100.0%**	**1.02 [0.88, 1.17]**
Heterogeneity: $Chi^2 = 31.18$, df = 9 (P = 0.0003); $I^2 = 71\%$				
Test for overall effect: Z = 0.23 (P = 0.81)				
Test for subgroup differences: $Chi^2 = 6.24$, df = 1 (P = 0.01), $I^2 = 84.0\%$				

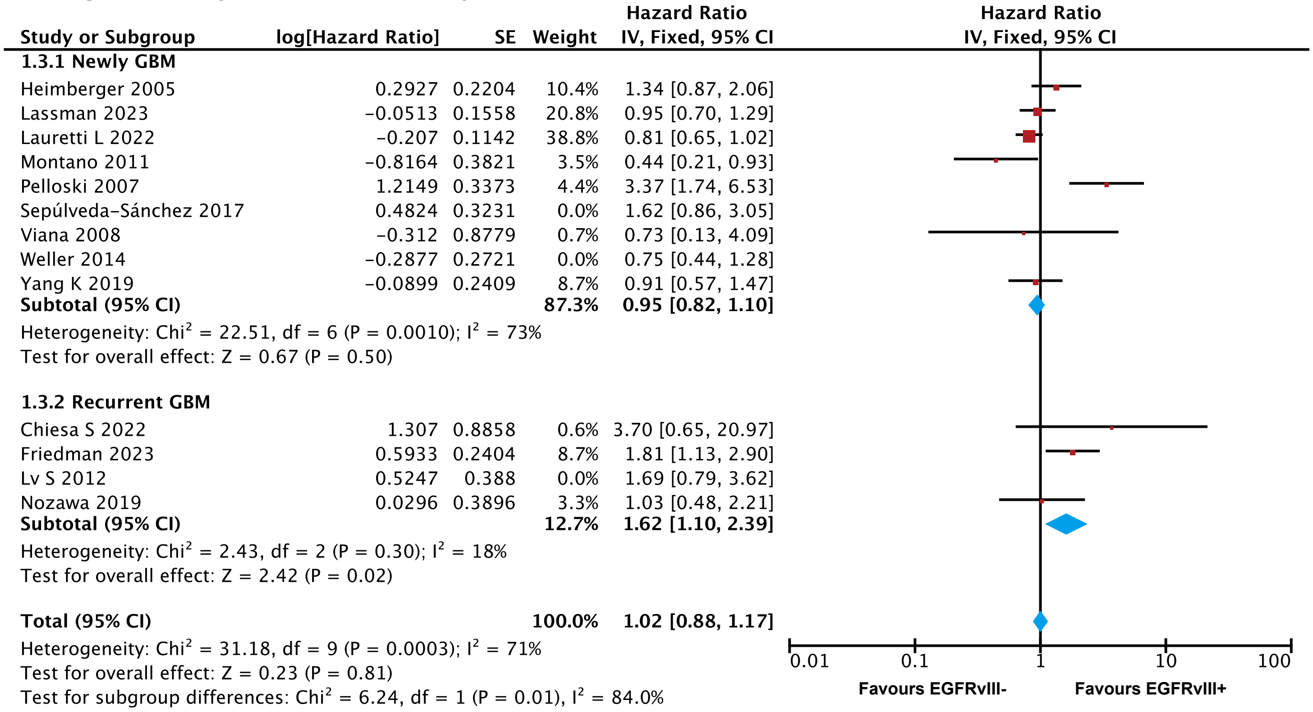

Figure S5. Reanalysis after excluding studies with IDH-mutant patients.
Random-effects model analysis of the impact of EGFRvIII mutation on OS (a) and PFS (b) in GBM patients. (c) Random-effects model analysis of the association between EGFRvIII mutation and OS in GBM patients from the Americas, Asia, or Europe. (d) Subgroup analysis of EGFRvIII mutation and OS in newly diagnosed or recurrent GBM patients using a fixed-effects model.

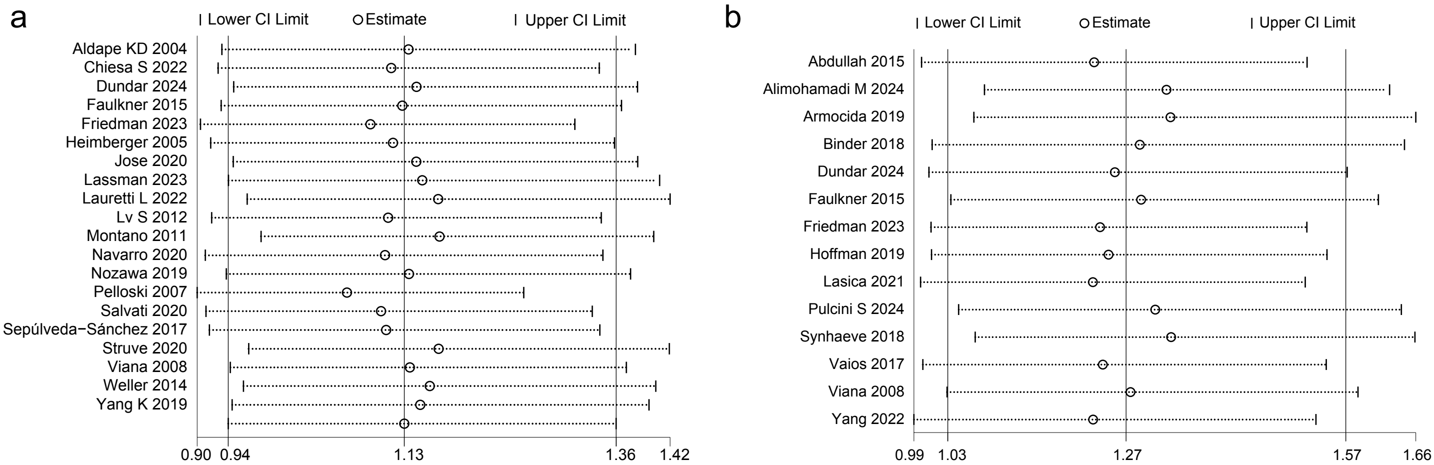

Figure S6. Sensitivity analysis using the leave-one-out approach to assess the robustness of the meta-analysis results. (a) Sensitivity analysis for the analysis to evaluate the association between EGFRvIII mutation and OS in GBM patients. (b) Sensitivity analysis for the analysis to evaluate the association between EGFR amplification and OS in GBM patients.

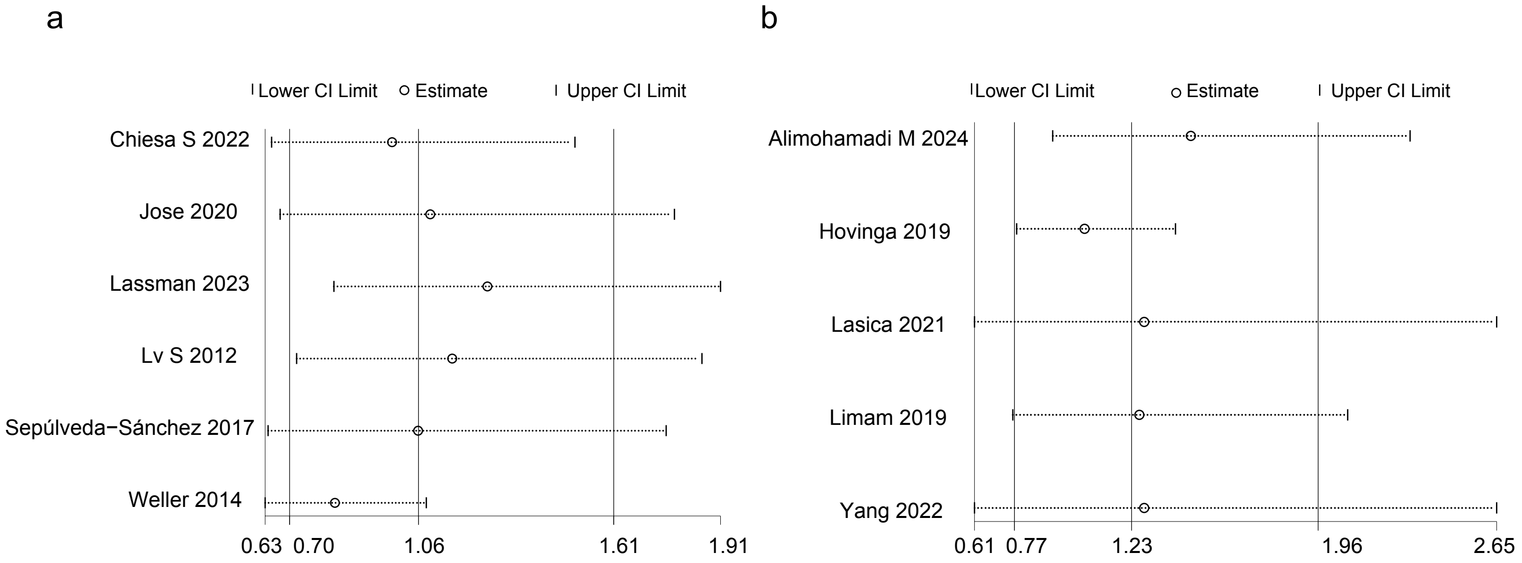

Figure S7. Sensitivity analysis using the leave-one-out approach to assess the robustness of the meta-analysis results.
(a) Sensitivity analysis for the analysis to evaluate the association between EGFRvIII mutation and PFS in GBM patients.
(b) Sensitivity analysis for the analysis to evaluate the association between EGFR amplification and PFS in GBM patients.

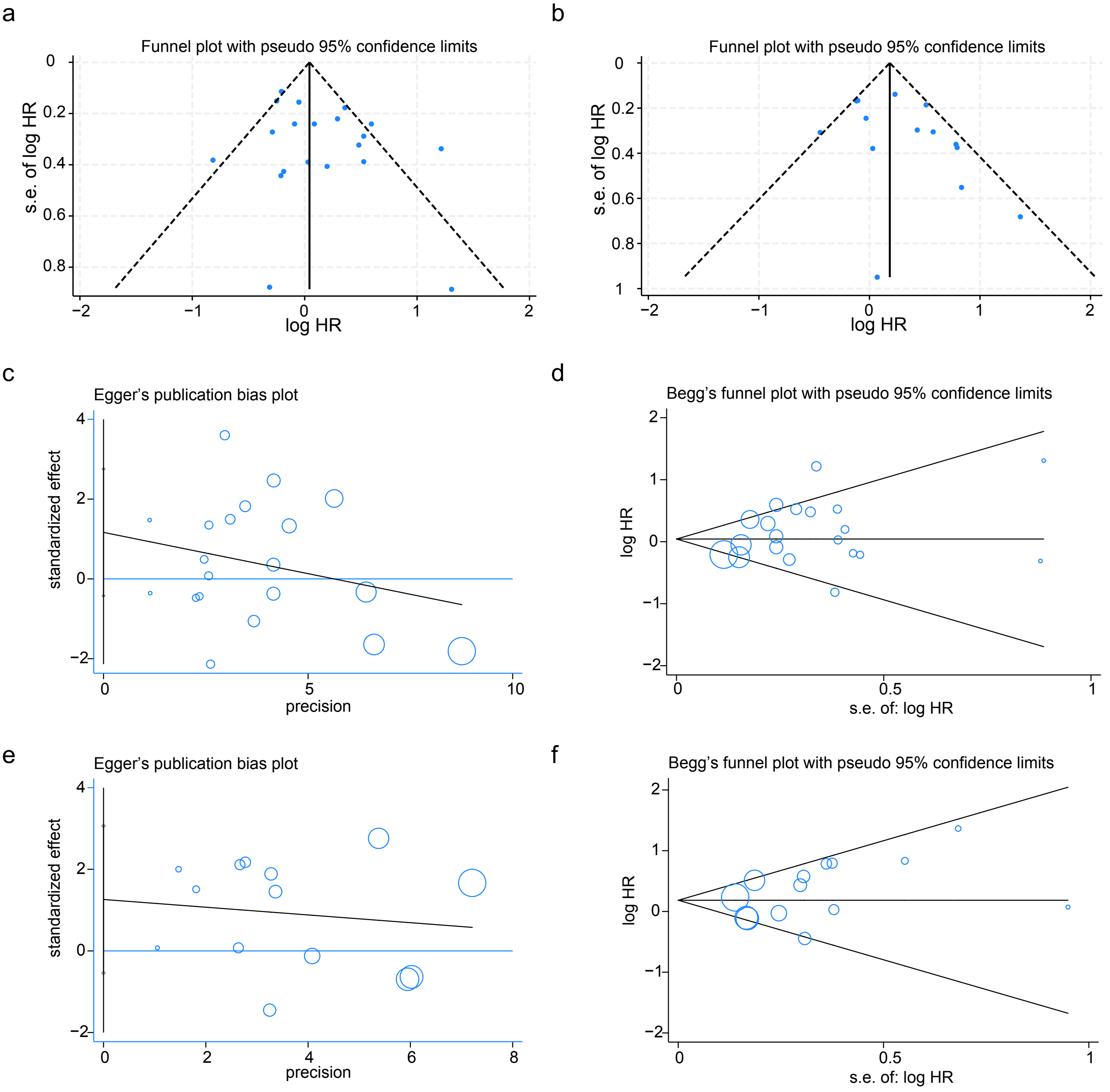

Figure S8. Funnel plots and publication bias analysis for the studies included in this meta-analysis.
(a) Funnel plot for the association between EGFRvIII mutation and OS in GBM patients.
(b) Funnel plot for the association between EGFR amplification and OS in GBM patients.
(c) Egger's test for publication bias in studies analyzing the association between EGFRvIII mutation and OS in GBM patients.
(d) Begg's test for publication bias in studies analyzing the association between EGFRvIII mutation and OS in GBM patients.
(e) Egger's test for publication bias in studies analyzing the association between EGFR amplification and OS in GBM patients.
(f) Begg's test for publication bias in studies analyzing the association between EGFR amplification and OS in GBM patients.
All analyses were performed using Stata 18.0.
